# Liposomal Formulation of Hydroxychloroquine Can Inhibit Autophagy In Vivo

**DOI:** 10.3390/pharmaceutics17010042

**Published:** 2024-12-30

**Authors:** Wieslawa H. Dragowska, Jagbir Singh, Mohamed Wehbe, Malathi Anantha, Katarina Edwards, Sharon M. Gorski, Marcel B. Bally, Ada W. Y. Leung

**Affiliations:** 1 Department of Experimental Therapeutics, BC Cancer, Vancouver, BC V5Z 1L3, Canada; visia.dragowska@gmail.com (W.H.D.); a_malthi@yahoo.com (M.A.); mbally@bccrc.ca (M.B.B.); 2 Department of Pathology and Laboratory Medicine, University of British Columbia, Vancouver, BC V6T 1Z7, Canada; 3 Faculty of Pharmaceutical Sciences, University of British Columbia, Vancouver, BC V6T 1Z3, Canada; 4 Department of Chemistry, Ångström Laboratory, Uppsala University, 751 20 Uppsala, Sweden; katarina.edwards@kemi.uu.se; 5 Canada’s Michael Smith Genome Sciences Centre, BC Cancer, Vancouver, BC V5Z 4S6, Canada; sgorski@bcgsc.ca; 6 Department of Molecular Biology and Biochemistry, Simon Fraser University, Vancouver, BC V5A 1S6, Canada; 7 NanoMedicines Innovation Network, Vancouver, BC V6T 1Z3, Canada; 8 Cuprous Pharmaceuticals Inc., Vancouver, BC V6T 1Z3, Canada

**Keywords:** liposomes, hydroxychloroquine, autophagy, breast cancer, gefitinib

## Abstract

**Background/Objectives:** Preclinical studies have shown that the anti-malarial drug hydroxychloroquine (HCQ) improves the anti-cancer effects of various therapeutic agents by impairing autophagy. These findings are difficult to translate in vivo as reaching an effective HCQ concentration at the tumor site for extended times is challenging. Previously, we found that free HCQ in combination with gefitinib (Iressa^®^, ZD1839) significantly reduced tumor volume in immunocompromised mice bearing gefitinib-resistant JIMT-1 breast cancer xenografts. Here, we sought to evaluate whether a liposomal formulation of HCQ could effectively modulate autophagy in vivo and augment treatment outcomes in the same tumor model. **Methods:** We developed two liposomal formulations of HCQ: a pH-loaded formulation and a formulation based on copper complexation. The pharmacokinetics of each formulation was evaluated in CD1 mice following intravenous administration. An efficacy study was performed in immunocompromised mice bearing established JIMT-1tumors. Autophagy markers in tumor tissue harvested after four weeks of treatment were assessed by Western blot. **Results:** The liposomal formulations engendered ~850-fold increases in total drug exposure over time relative to the free drug. Both liposomal and free HCQ in combination with gefitinib provided comparable therapeutic benefits (*p* > 0.05). An analysis of JIMT-1 tumor tissue indicated that the liposomal HCQ and gefitinib combination augmented the inhibition of autophagy in vivo compared to the free HCQ and gefitinib combination as demonstrated by increased LC3-II and p62/SQSTM1 (p62) protein levels. **Conclusions:** The results suggest that liposomal HCQ has a greater potential to modulate autophagy in vivo compared to free HCQ; however, this did not translate to better therapeutic effects when used in combination with gefitinib to treat a gefitinib-resistant tumor model.

## 1. Introduction

The U.S. breast cancer statistics indicate that one in eight women will develop invasive breast cancer during their lifetime [1]. While successes in the diagnosis and treatment of breast cancer have led to dramatic improvements in survival rates over the last few decades, advanced metastatic breast cancer still presents as an incurable disease with limited treatment options due to intrinsic or acquired resistance to previously used therapies [2,3,4,5]. Over the past decade, there has been a growing interest in macroautophagy (referred to herein as autophagy), a crucial cell survival mechanism contributing to therapeutic resistance in cancer, and pre-clinical studies have demonstrated that inhibiting autophagy augments the efficacy of various anti-cancer therapeutics [6,7,8,9,10,11,12,13,14]. The physiological role of autophagy is to remove dysfunctional cellular components and to withstand energy stress [15,16,17]. It is a dynamic process referred to as autophagic flux, which is initiated by the development of phagophores engulfing defective cellular organelles and cytoplasmic constituents. These structures mature into vesicular double-membrane autophagosomes that deliver their cargo to the lysosome for degradation by hydrolytic enzymes, a process that generates recyclable monomeric molecules (e.g., amino acids) and energy for the stressed cells [15,16,17,18]. Evidence provided by in vitro studies supports the notion that autophagy is exploited by cancer cells to enhance survival during stress inflicted within the microenvironment (e.g., hypoxia), as well as by anti-cancer treatments such as radiotherapy and chemotherapy using conventional cytotoxics and targeted agents [11,19]. In breast cancer, targeted agents have become important therapeutic tools; however, the development of resistance to these agents presents a significant problem in the clinic [20,21]. This is exemplified by the therapeutic antibody trastuzumab that interferes with human epidermal growth factor receptor 2 (HER2) function, the dual epidermal growth factor receptor (EGFR) and the HER2 inhibitor lapatinib, as well as the EGFR inhibitor gefitinib [6,7,22]. In vitro studies showed that resistance to EGFR and HER2 inhibitors is, in part, attributed to cytoprotective autophagy, and that inhibiting this process with genetic or pharmacological inhibitors, such as hydroxychloroquine (HCQ), promotes the sensitization of breast cancer cells to treatments [20,22].

HCQ is an approved anti-malarial drug that has the potential to be repurposed as an autophagy inhibitor and is currently undergoing clinical trials for different cancer indications, including breast cancer [13,20,23,24,25,26]. HCQ inhibits the late stage of autophagic flux, primarily by impairing autophagosome–lysosome fusion and by reducing the acidification of the lysosomes needed for the activation of hydrolytic enzymes [15,27,28]. Various preclinical studies showed that HCQ can sensitize breast cancer cells to classical chemotherapeutics and targeted agents [7,10,29]; however, only a moderate tumor growth inhibition was achieved in vivo. Results from phase I/II clinical trials of oral HCQ in combination with various anti-cancer modalities also did not report improved outcomes [13,25,30,31]. Perhaps this is not unexpected, since the HCQ concentration required to achieve a significant inhibition of autophagy in vitro was ~20 µM [7]. Further, this drug concentration needed to be maintained over several days. It is unrealistic to expect that such levels could be achieved over an extended time when using the currently approved oral HCQ dosage form. Specifically, when HCQ is given orally in humans, the highest plasma concentrations achievable are 1–3 µM [32]. To date, over 1000 studies have looked at HCQ as an autophagy inhibitor, and this includes over 50 clinical trials, but only a dozen studies have contemplated drug delivery approaches [33,34,35]. The potential for the reformulation of HCQ for clinical applications is great, but this work is hindered by studies that have been completed with HCQ given orally without therapeutic benefit and the challenges of developing complicated formulations that may be better designed to address the ability for HCQ to improve treatment outcomes.

To increase the activity of HCQ in vivo, higher concentrations in plasma and tumor tissue for extended periods are required, in addition to reconsidering the dosage form and the route of administration. This could potentially be accomplished by utilizing a simple, easy-to-manufacture liposomal formulation of HCQ that would increase plasma levels of the drug over extended time periods and facilitate greater levels of HCQ delivery to site(s) of tumor growth, while at the same time limiting the delivery of HCQ to cardiac tissue. Numerous studies have shown that liposomal formulations of selected agents alter the agents’ pharmacokinetics (PK) characteristics following IV administration [36,37,38,39,40], resulting in significant increases in the liposomal agent’s circulation time and concentration in plasma. These changes also correlated with an increased accumulation of the associated agents in sites that exhibit altered blood vessel structures, such as sites of tumor growth, while reducing agent delivery to sites where toxicities are seen. In aggregate, liposomal formulations, depending on the agent that has been formulated, can improve efficacy and reduce toxicity [41,42,43,44,45,46,47,48,49].

For the studies reported here, liposomal HCQ formulations were prepared using two different loading techniques to encapsulate HCQ into liposomes. Encapsulation was achieved through the use of a transmembrane pH gradient (inside acid) [50] and/or HCQ–metal complexation [23,51]. The liposomal HCQ formulations developed for this study used preformed liposomes composed of ammonium sulfate or copper sulfate, and the liposomal lipid composition was 1,2-Distearoyl-sn-glycero-phosphocholine (DSPC) and cholesterol (Chol) (55:45 mol%). Following IV administration of the HCQ containing liposomes, a pharmacokinetics analysis measuring the area under the curve from 0 to infinity (AUC_0-∞_) showed that the liposomal HCQ formulations engendered an ~850-fold increase in total drug exposure over time compared to a free HCQ given IV. The method of encapsulation made little difference in the plasma HCQ elimination results obtained; however, the efficacy studies focused on the use of the copper complexation method, in part because of recent results highlighting that copper can influence a number of cell death mechanisms including apoptosis, autophagy and its own unique form of cell death referred to as cuproptosis [52,53,54,55,56,57,58]. Intravenous liposomal HCQ or free HCQ given intraperitoneally (IP) was assessed in combination with the autophagy-inducing EGFR inhibitor gefitinib in immunocompromised mice bearing established gefitinib-resistant JIMT-1 breast cancer xenografts. The results suggest that although autophagic markers in tumor tissue harvested after 4 weeks of treatment engendered a more potent inhibition of gefitinib-induced autophagy in vivo by the selected liposomal HCQ formulation versus free HCQ, these molecular changes did not result in a significant difference in treatment outcomes. It is important to note that in the studies reported here, the free drug was given IP and was dosed more frequently (due to the fact that IP administration can be given daily, but IV administration can only be performed every other day); thus, the free drug was given at a 60% higher total cumulative dose when compared to the liposomal drug.

## 2. Materials and Methods

### 2.1. Chemicals

Hydroxychloroquine Sulfate (HCQ) was purchased from Acros Organic (Newark, NJ, USA). 1,2-Distearoyl-sn-glycero-phosphocholine (DSPC) and cholesterol (Chol) were obtained from Avanti Polar Lipids (Alabaster, AL, USA). ^3^H-cholesteryl hexadecyl ether (^3^H-CHE) was purchased from Perkin-Elmer Life Sciences (Boston, MA, USA). Calcium Ionophore A23187 (Calcimycin) was sourced from Sigma (Oakville, ON, Canada). All other chemicals used were of analytical grade. Gefitinib was purchased from LC Laboratories (Woburn, MA, USA).

### 2.2. Liposome Preparation

DSPC/Chol (55:45 mol:mol) liposomes were prepared using a thin-film hydration technique as described previously [24]. Briefly, lipids were dissolved in chloroform at the required molar ratio and labeled with the nonexchangeable and nonmetabolizable lipid marker ^3^H-CHE and dried to a thin film under N_2_ gas. The lipid film produced was further dried under vacuum for 2 h to remove any residual solvent. The lipid films were hydrated with 250 mM (NH_4_)_2_SO_4_ buffer (pH 5.1) or unbuffered 300 mM CuSO_4_ (pH = 3.5) at 65 °C for three hours, followed by five freeze–thaw cycles in liquid nitrogen and a 65 °C water bath. Finally, the resulting multilamellar liposomes were extruded (Extruder™, Evonik, Burnaby, BC, Canada) through stacked 0.1 µm and 0.08 µm polycarbonate filters 10 to 15 times at 65 °C to obtain unilamellar liposomes with a mean particle size of 100 ± 20 nm, as determined using the Phase Analysis Light-Scattering technique (ZetaPALS, Brookhaven Instruments Corp., Holtsville, NY, USA). The external buffer of (NH_4_)_2_SO_4_-liposomes was exchanged to phosphate buffer saline (PBS, pH 7.4) using Sephadex G-50 size exclusion chromatography. The CuSO_4_-liposomes were first processed through a size exclusion column prepared with Sephadex G-50 and equilibrated with SHE buffer (300 mM sucrose, 20 mM HEPES, 15 mM EDTA, pH 7.4) to remove unencapsulated copper. The external SHE buffer was then exchanged to PBS (pH 7.4) using the same method.

### 2.3. Cu-Gluconate Liposomes and Sucrose–HEPES Liposomes

To determine the effect of the pH gradient on HCQ encapsulation, DSPC/Chol (55:45 mol ratio) lipid films were hydrated with sucrose–HEPES (SH) buffer (pH 7.4) and extruded as described above, with the final elution in SH buffer itself. The second set of liposomes was prepared with 100 mM of copper gluconate solution (pH 7.4), such that any encapsulation would be due to copper complexation.

### 2.4. Drug Loading

The HCQ loading rates were determined by incubating HCQ with preformed DSPC/Chol liposomes at different temperatures at an initial HCQ-to-liposomal lipid molar ratio of 0.25 unless indicated otherwise. At defined time points, an aliquot of 80 µL was passed through 1 mL Sephadex G-50 spin columns equilibrated with an equal volume of PBS buffer to remove the encapsulated HCQ. The eluted fractions were analyzed for HCQ and lipid concentrations. HCQ was determined by measuring UV absorbance at 330 nm (Multiskan Spectrum, Thermo Scientific, Waltham, MA, USA), and lipid concentration was estimated by measuring ^3^H-CHE using the liquid scintillation counting method (LS6500 Multipurpose Scintillation Counter; Beckman Coulter, Mississauga, ON, Canada).

### 2.5. Cryo-Transmission Electron Microscopy (Cryo-TEM)

For cryo-TEM images, non-radioactive liposomes were prepared as described before, and HCQ was encapsulated by mixing free HCQ with the liposomes at 50 °C for 10 min. Samples were then concentrated using tangential flow filtration. Subsequently, 1–2 μL of the liposomal sample (~10 μM liposomal lipid) was placed onto a copper grid coated with cellulose acetate butyrate polymer film and blotted using a filter paper to remove excess liquid. The sample was then flash-frozen in liquid ethane and transferred to liquid N_2_ to maintain the temperature below −165 °C. The samples were analyzed using a Zeiss LIBRA-120 transmission electron microscope (Carl Zeiss Inc., Oberkochen, Germany) under zero-loss bright-field mode and an accelerating voltage of 80 kV.

### 2.6. In Vitro Liposomal Formulation Stability

An aliquot of 400 µL of liposomal HCQ formulations, prepared at a drug-to-lipid ratio of 0.2:1 (mol/mol), was placed into dialysis bags (Spectra/Por^®^, MWCO 12–14 kD, Spectrum Laboratories, Piscataway, NJ, USA) and dialyzed against 1 L of PBS buffer maintained at 37 °C. At the indicated time points, the dialysis bag was removed, and an aliquot of the sample was eluted through 1 mL equilibrated Sephadex G-50 spin columns to remove unencapsulated HCQ. The drug release was quantified by measuring the HCQ-to-liposomal lipid ratio in the sample at different time points. Liposome-associated HCQ was measured using UV–Vis spectroscopy, and the lipid concentration was measured using liquid scintillation counting as described above.

### 2.7. High-Content Analysis of Viability and Autophagy

MCF7 cells were stably transfected with the enhanced green fluorescent protein (EGFP)–microtubule-associated protein 1 light chain 3B (MAP1LC3B) construct to generate MCF7-GFPLC3 cells as described previously [59]. The SKBR3 cells were from the American Type Culture Collection (ATCC). MCF7-GFPLC3, SKBR3 and JIMT-1 cells were maintained in RPMI, McCoy’s 5A and in DMEM, respectively, supplemented with 10% FBS. Cells were seeded in 96-well plates in triplicate wells and, after overnight adhesion, treated for 24 to 72 h with the vehicle (PBS), free or liposomal HCQ and drug-free liposomes. Liposomal HCQ or drug-free liposomes at equivalent liposomal lipid concentrations were diluted to the desired HCQ concentration with PBS, and 10× concentrated agents were added to the cells in culture media supplemented with 10% FBS, such that the desired final HCQ concentration could be achieved. At specified time points, the MCF7-GFPLC3 cells were stained in situ with Hoechst 33,342 (stains the nuclei of viable and dead cells) and ethidium homodimer-I (stains the nuclei of dead cells), and SKBR3 cells were stained with DRAQ5 (stains the nuclei of viable and dead cells), ethidium homodimer-I and monodansylcadaverine (MDC) (stains autolysosomes). The plates were then imaged with an IN Cell Analyzer 1000 (GE Healthcare, Chicago, IL, USA), and the imaging data were analyzed with high-content analysis (HCA) Investigator^TM^ software v 1.0 (GE Healthcare), permitting a quantitative analysis of viability, along with the GFP- or MDC-positive cellular organelle content expressed as the total organelle area/cell (TOA).

### 2.8. Western Blot Analysis

Cells were seeded in 6 cm diameter dishes and treated as described above. Cell lysates were obtained by scraping cultured cells in RIPA buffer containing protease inhibitors (Complete-Mini protease inhibitor tablets, Boehringer Mannheim GmBH, Mannheim, Germany) and phosphatase inhibitors (PhosSTOP tablets: Roche, Basel, Switzerland). Tumor and organ tissues harvested from animals were homogenized over two 30 s cycles with the MP-Fastprep Homogenizer (MP Biomedicals, Santa Ana, CA, USA) in cold RIPA buffer (see above). The cell and tissue lysates were then incubated on ice for 5 min and centrifuged at 15,000× *g* for 20 min, and the supernatants were stored in −80 °C. The protein content in the lysates was determined using the Bicinchoninic Acid (BCA) Assay (Pierce™ BCA Protein Assay Kit, Thermo Fisher Scientific). Equivalent amounts of protein from each tumor or organ tissue were pooled for each treatment group. For cell and tissue lysates, 17–30 µg and 50 µg of total protein per sample, respectively, was separated on 4–12% Bis-Tris gels (Novex, Life Technologies, Carlsbad, CA, USA) and then transferred onto nitrocellulose membranes (BioRad, Hercules, CA, USA, trans-blot turbo mini 0.2 µm nitrocellulose membranes). The membranes were blocked with 5% skim milk for 1 h at room temperature. Before staining with the corresponding antibodies, the membranes were cut according to the molecular weight of the proteins of interest, and the corresponding membrane strips were stained overnight in their respective antibody solutions at 4 °C. The following antibodies were used: LC3B (Abcam, Cambridge, UK, cat#48394), SQSTM1/P62 (Cell Signaling Technology, Danvers, MA, USA, cat#5114; Santa Cruz Biotechnology, Dallas, TX, USA, cat#sc-28359), β-actin (Sigma-Aldrich, cat#A5441) and β-tubulin (Abcam, cat#6046). The horseradish peroxidase-conjugated secondary antibody was from Promega, and the prestained protein ladder was from GeneDireX BLUeye, Hsinchu, Taiwan (cat#PM007-0500). Protein bands were detected by enhanced chemiluminescence (Clarity Western ECL Substrate, Bio-Rad) and visualized with the ChemiDoc MP System (Bio-Rad). Images were saved in SCN format using Image Lab 5.1 software (Bio-Rad). A densitometric analysis was performed on non-saturated protein bands using the Image Lab 5.1 software to approximate the relative protein expression across various samples normalized to the respective loading control. Each Western blot analysis was repeated 2–3 times for consistency for each set of samples.

### 2.9. HCQ and Liposomal HCQ PK Study

PK studies were conducted in female CD1 mice (18–23 g; 6–8 weeks; n = 4 per time point per group). Selected liposomal HCQ formulations or free HCQ dissolved in saline were administered at 25 mg/kg. A single intravenous bolus injection was administered via the tail vein, and blood was collected by cardiac puncture after euthanizing the mice. Plasma was separated by centrifugation (2500 rpm for 15 min) and stored at −80 °C for subsequent analysis. HCQ was measured using a Waters Alliance HPLC Module 2695 (Milford, CT, USA) and a multi-wavelength fluorescence detector 2475 (Milford, CT, USA) at λ_ex_ = 335 nm and λ_em_ = 450 nm. A Waters Symmetry RP C_18_ column (5 µm, 100 Å, 4.6 mm × 250 mm) was used to separate HCQ using a mixture of mobile-phase methanol/phosphate buffer (10 mM, pH 7.4) at 45:55%. The chromatography column was heated to 50 °C while samples were maintained at 4 °C. Plasma samples were mixed with methanol and centrifuged at 14,000 rpm for 10 min to remove precipitated plasma proteins. Then, sample supernatants were appropriately diluted and injected into the column. A flow rate of 1.2 mL/min was used for a total run time of 9.5 min.

### 2.10. Efficacy Study

For efficacy studies, 7.5 × 10^6^ JIMT-1 (gefitinib-resistant) cells from the German Collection of Microorganisms and Cell Culture (Deutsche Sammlung von Mikroorganismen und Zellkulturen GmbH (DSMZ), Braunschweig, Germany) were cultured in DMEM 10% FBS and injected subcutaneously (s.c.) on the back of female 129S6/SvEvTac-*Rag2^tm1Fwa^* immunocompromised mice. Animals with established tumors (~100 mm^3^) were then randomized to different treatment groups (6–8 animals per group). Mice were left untreated or dosed with the indicated agents for 4 weeks. Gefitinib was prepared in 0.5% Tween-80 in sterile milli-Q water and administered at 100 mg/kg as an oral gavage QDx5 (Monday to Friday) divided into two doses given 30–60 min apart. Since gefitinib was dosed orally, it was not possible to consider giving HCQ orally under the animal care protocol approved at that time. While this was done previously (dosing separated by 4 h), the animal care committee and care staff were very concerned about damage to the esophagus caused by twice daily oral gavage. As an alternative, free HCQ, prepared in saline, was administered IP at 60 mg/kg QDx5 (Monday to Friday). The liposomal HCQ formulation used (the one based on the copper complexation method) was administered IV at a dose of 60 mg/kg. The liposomal formulation was designed to provide a formulation suitable for IV administration and was expected to increase HCQ blood and tissue levels when compared to free HCQ given by other administration routes. Due to potential tail vein damage caused by repeat dosing, the liposomal formulation was only dosed 3 times per week (Monday, Wednesday and Friday). From a cumulative dosing perspective, this study was biased against the liposomal HCQ formulation as it was administered at a 40% lower total cumulative dose when compared to free HCQ. In the groups treated with two drugs, gefitinib was dosed first, followed 1 h later by free HCQ (IP) or liposomal HCQ (IV). All formulations were prepared weekly and kept at 4 °C prior to administration. Tumor volume was calculated by the formula (L × W^2^)/2 and expressed as a change in tumor volume relative to the volume measured on the first day of treatment, expressed as 100%. This step was applied to account for different tumor volumes when treatment was initiated. Four hours after the last dose, tumors and livers were harvested, flash-frozen in liquid nitrogen and stored at −80 °C for later processing with Western blotting. During the study, animals were monitored for body weight loss and other signs of sickness due to treatment-related side effects or tumor burden.

### 2.11. Ethics Statement

All animal studies and procedures were performed following protocols (A22-0274) approved by the Institution Animal Care Committee at the University of British Columbia (Vancouver, BC, Canada). These studies met the guidelines of the Canadian Association of Animal Care.

### 2.12. Statistics

The statistical analyses were performed using GraphPad Prism 6. Statistical analyses comparing tumor volumes on the last day of treatment were performed using a two-way analysis of variance (ANOVA) followed by Holm Sidak’s multiple comparisons test. A *p*-value < 0.05 was considered statistically significant.

## 3. Results

### 3.1. HCQ Loading into Liposomes Depends on Transmembrane pH Gradient

**Figure 1:**
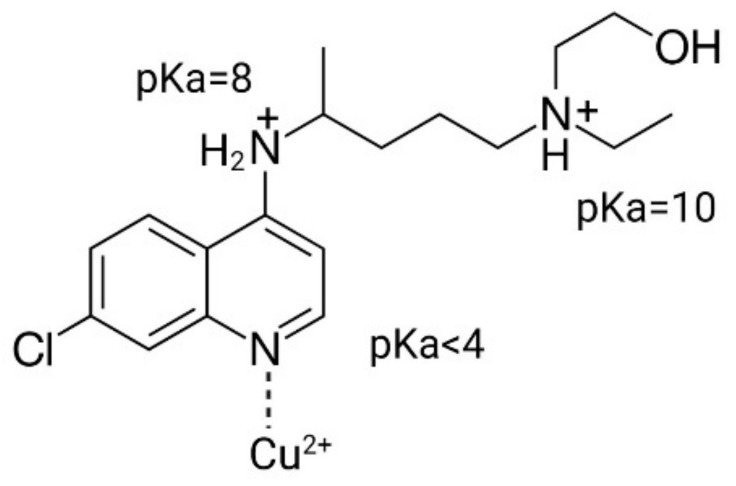
Chemical structure of hydroxychloroquine. Dash line indicates where the Cu^2+^ ion coordinates with the compound to form a Cu(II)-HCQ complex.

**Figure 2:**
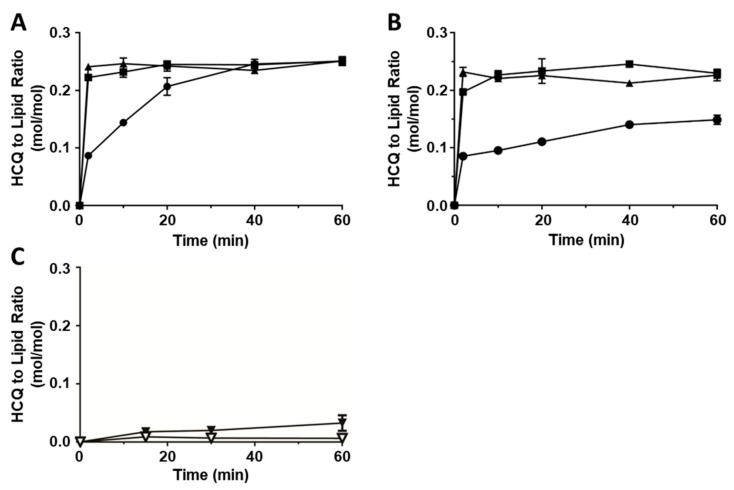
Remote loading of HCQ into preformed liposomes containing (NH_4_)_2_SO_4_ or CuSO_4_ depends on a transmembrane pH gradient (**A**) HCQ loading into (NH_4_)_2_SO_4_-containing liposomes (pH 5.1) at 20 °C (●), 40 °C (■) and 50 °C (▲). (**B**) HCQ loading into CuSO_4_-containing liposomes (pH 3.5) at 20 °C (●), 40 °C (■) and 50 °C (▲). (**C**) HCQ loading into liposomes with internal SH buffer pH 7.4 (▽) or 100 mM Cu-gluconate pH 7.4 (▼) at 40 °C. Data are plotted as mean ± SEM (n = 3). If error bars are not indicated; it is because the error was within the size of the symbol used.

Based on the presence of a protonizable amine group as well as the presence of copper-coordinating atoms in its chemical structure (Figure 1), two methods were suitable to encapsulate HCQ into liposomes. The first method involved adding HCQ to liposomes prepared in (NH_4_)_2_SO_4_ buffer (pH 5.1) to generate a transmembrane pH gradient (see Section 2.2) [46,47,60], and the second method involved adding HCQ to liposomes prepared in unbuffered CuSO_4_ (pH 3.5), where HCQ encapsulation was facilitated through formation of Cu(II)-HCQ complexes [23,61]. The results summarized in Figure 2A show that adding HCQ to the preformed (NH_4_)_2_SO_4_-containing liposomes resulted in an efficient (>98% of the added drug) encapsulation of HCQ achieved in 40 min at 20 °C. When the incubation temperature was increased to 40 °C or 50 °C, the encapsulation was complete by 3 min. For formulations prepared using preformed liposomes with unbuffered CuSO_4_ (pH 3.5) (Figure 2B), the loading efficiency achieved at 20 °C was only about 60% even after 60 min of incubation. The rate of HCQ loading increased when the incubation temperature was elevated to 40 °C or 50 °C. At these temperatures, the encapsulation of HCQ was rapid, and >98% of the added HCQ was encapsulated within 3 min (Figure 2B). Control liposomes were prepared: (i) liposomes prepared in SH buffer (pH 7.4) or (ii) liposomes prepared with 100 mM copper gluconate (pH 7.4). These liposomes were mixed with HCQ and incubated for 60 min at 40 °C (Figure 2C). The liposomes prepared with SH buffer (pH 7.4) (see ▽) exhibited HCQ-to-lipid ratios indicating <5% encapsulation efficiency even after 60 min. Liposomes prepared with Cu-gluconate (pH 7.4) also showed <10% HCQ association after a 60 min incubation at 40 °C (see ▼). It should be noted that CuSO_4_ solutions cannot be prepared at a neutral pH due to the formation of copper hydroxide precipitates. For many copper complexing compounds, liposomes with 100 mM of encapsulated copper gluconate (adjusted to pH 7.4) can serve as a control to demonstrate that encapsulation is due to copper complexation. However, this approach was not appropriate for a weak copper binder. The results suggest that HCQ was not able to displace gluconate from copper in these studies. Thus, while the liposomes with unbuffered CuSO_4_ (pH 3.5) could be used to efficiently encapsulate HCQ, it was not possible to distinguish between the role of the internal pH (3.5) and internal Cu. In aggregate, these studies demonstrate that HCQ can be encapsulated efficiently inside liposomes containing (NH_4_)_2_SO_4_ or CuSO_4_. The presence of a transmembrane pH gradient is an important factor driving the encapsulation of HCQ.

### 3.2. Characterization of Liposomes with Encapsulated HCQ

**Figure 3:**
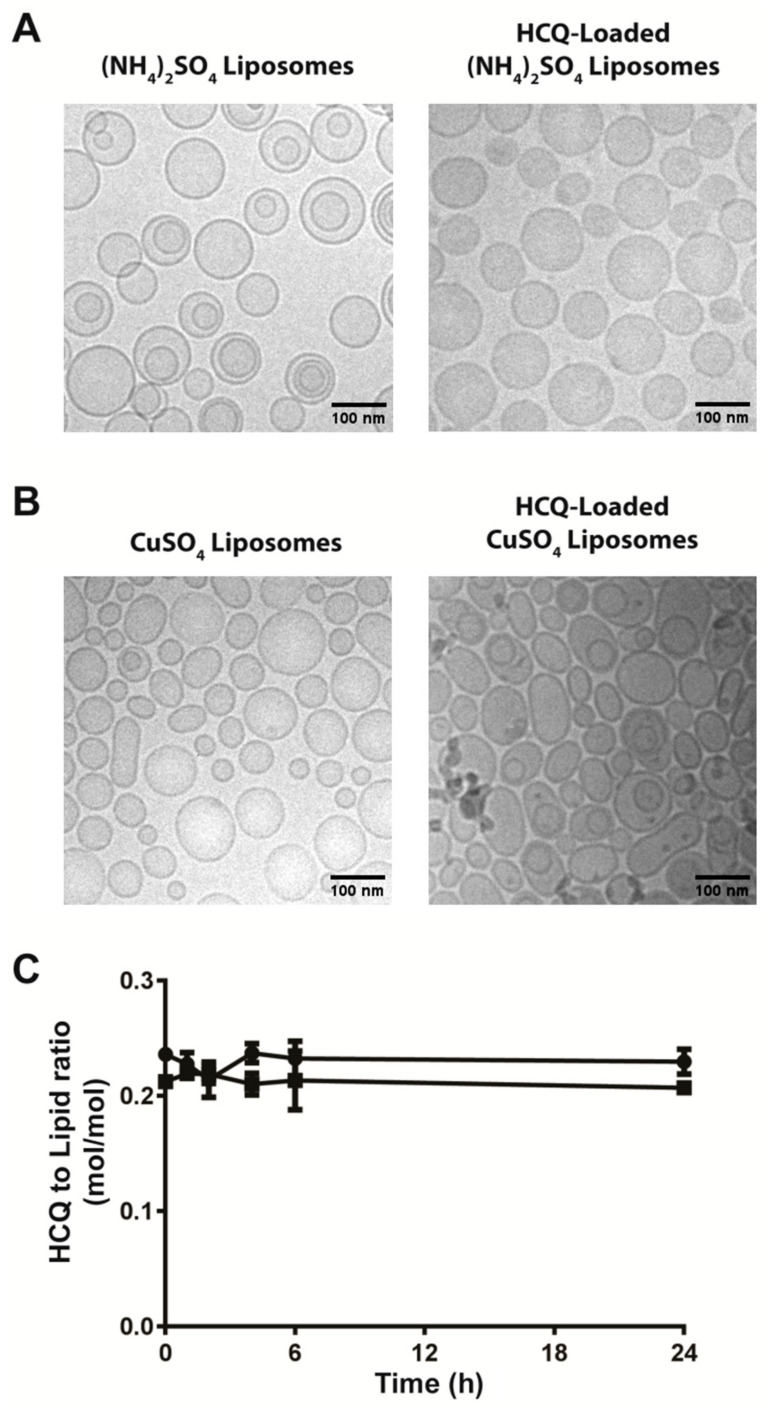
Characterization of HCQ liposomes loaded by (NH4)_2_SO_4_ or CuSO_4_ gradients. (**A**,**B**) Cryo-TEM images of empty (**left**) and HCQ-loaded (**right**) liposomes using pH transmembrane gradients formed by 250 mM (NH_4_)_2_SO_4_ (**A**) or 300 mM CuSO_4_ (**B**). (**C**) Retention of HCQ in CuSO_4_ (■) or (NH_4_)_2_SO_4_ (●)—loaded liposomes assessed at 37 °C (see Section 2). The (**C**) data are plotted as mean ± SEM (n = 3). If error bars are not indicated; it is because the error was within the size of the symbol used.

The HCQ liposome formulations prepared were characterized to assess liposome size and appearance. Cryo-transmission electron microscopy images of the (NH_4_)_2_SO_4_ and CuSO_4_ liposomes were taken prior to HCQ encapsulation (Figure 3A,B, left images) and after HCQ encapsulation (Figure 3A,B, right images). These images show that the liposomes appeared similar regardless of whether the HCQ was encapsulated or not. Table 1 summarizes the characteristics of the liposomes before and after HCQ encapsulation. The particle size and polydispersity of the formulations did not change as a function of HCQ encapsulation. The stability of the liposomal HCQ formulations was assessed over 24 h in vitro by dialyzing the HCQ formulations against a 1000-fold excess of PBS at 37 °C and quantifying the drug release. After 24 h, the (NH_4_)_2_SO_4_ and CuSO_4_ HCQ liposomal formulations retained >95% of the loaded HCQ, as determined by measurements of the HCQ-to-lipid ratio (Figure 3C). Further, if the liposomal formulations were stored for 30 days at 4 °C, there would be no change in liposome size, polydispersity index or HCQ-to-lipid ratio.

### 3.3. In Vitro Inhibition of Autophagy as a Surrogate Measure of HCQ Release from Liposomes

**Figure 4:**
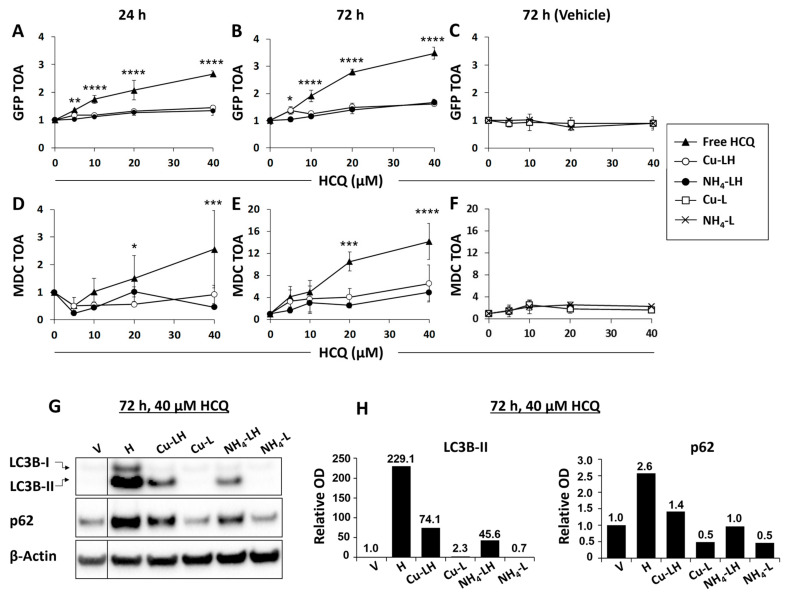
Inhibition of autophagy as a surrogate measure of HCQ release from (NH_4_)_2_SO_4_ and CuSO_4_-loaded liposomes in vitro. (**A**–**F**) Autophagy levels measured by high-content analysis (HCA) in MCF7-GFPLC3 (**A**–**C**) and SKBR3 (**D**–**F**) breast cancer cells in the presence of free or liposomal HCQ. Cells were treated with the indicated agents for 24 (**A**,**D**) or 72 h (**B**,**C**,**E**,**F**) and then stained in situ with Hoechst33342 (MCF7-GFPLC3) or DRAQ5 and MDC (SKBR3). An increase in GFPLC3- or MDC-positive vesicles measured as total organelle area per cell (TOA) indicates the accumulation of autophagy-associated organelles. The TOA increase in treated cells was normalized to the TOA in untreated cells expressed as 1. Data (**A**–**F**) are plotted as mean ± SD (n = 3). Statistical analysis was performed via two-way ANOVA with Tukey multiple test corrections. * *p* < 0.05, ** *p* < 0.01, *** *p* < 0.001, **** *p* < 0.0001. The asterisks indicate statistically significant differences between the free HCQ and the two liposomal HCQ groups in (**A**,**B**,**E**). The asterisks in (**D**) represent statistically significant differences between free HCQ and Cu-LH groups. (**G**) Western blot analysis of LC3B-I/II levels in lysates derived from MCF7-GFPLC3 cells treated with free or liposomal HCQ. An increase in LC3B-II and p62 indicates an accumulation of autophagic organelles. β-Actin was used as a loading control. V: vehicle (PBS); H: free HCQ; Cu-LH: HCQ-loaded CuSO_4_ liposomes; Cu-L: drug-free CuSO_4_ liposomes; NH_4_-LH: HCQ-loaded (NH_4_)_2_SO_4_ liposomes; NH_4_-L: drug-free (NH_4_)_2_SO_4_ liposomes. The line between the vehicle (V) and HCQ (H) lanes indicates the spliced fragments originating from the same gel. Representative images of Western blots are shown. (**H**) The expression levels of LC3B-II and p62 proteins, as shown in (**G**), were measured by densitometry and normalized to the vehicle control expressed as 1.

To gain insight into whether the HCQ release from liposomes at 37 °C was functionally significant, an in vitro cell-based assay was performed in culture media supplemented with 10% FBS. For these studies, MCF7 cells expressing the GFPLC3 fluorescent protein (MCF7-GFPLC3) that incorporates into the autophagosomal membrane [7,15,16,62] and SKBR3 cells were used. The cells were treated for up to 72 h with free or liposomal HCQ up to 40 μM. When cells were treated with 80 μM free HCQ, significant cell toxicity was noted. Before imaging, the SKBR3 cells were labeled with acidotropic MDC dye that accumulates in autophagy-associated organelles [7,15]. The cells were then imaged in situ with the IN Cell 1000 HCA platform (see Section 2). The average total GFP- or MDC-positive organelle area per cell (TOA) was measured in the MCF7-GFPLC3 and SKBR3 cells, respectively. The data show that free HCQ triggered a dose- and time-dependent accumulation of autophagic organelles in the cells (Figure 4A,B,D,E). Twenty-four hours incubation with HCQ formulated in (NH_4_)_2_SO_4_ or CuSO_4_ liposomes engendered only a minor increase in TOA in the MCF7-GFPLC3 cells and did not increase TOA in the SKBR3 cells (Figure 4A,D) versus the significantly higher (*p* < 0.05) TOA present in cells incubated with free HCQ. After 72 h, a modest, dose-dependent increase in TOA was observed in MCF7-GFPLC3 and SKBR3 cells treated with liposomal HCQ, suggesting that there was some release of HCQ from the liposomes in culture (Figure 4B,E); however, these effects were significantly lower (*p* < 0.05) in MCF7-GFPLC3 cells for all tested concentrations of liposomal HCQ (Figure 4B) and for 20–40 µM HCQ in SKBR3 cells (Figure 4E) compared to the effects noted following treatment with free HCQ. Control liposomes prepared with (NH_4_)_2_SO_4_ or CuSO_4_ did not engender any noteworthy autophagy modulation (Figure 4C,F) when added at a lipid concentration that was equivalent to what was used with the formulated HCQ.

The effects of HCQ on autophagy in MFC7-GFPLC3 cells were also monitored by Western blot analysis of LC3B-II and sequestrome 1 (p62/SQSTM1 (p62)) protein levels. During the process of autophagy, LC3B protein is cleaved to generate LC3B-I and then conjugated to phosphatidylethanolamine (PE), resulting in the formation of lipidated LC3B-II [15]. The LC3B-II form is an integral part of the autophagosomal membrane, and its levels can be considered proportional to autophagosome content in cells [15]. The p62 protein is known to facilitate the autophagic degradation of ubiquitinated protein aggregates, and its levels serve as an indication of autophagic flux activity [15]. As both the induction and inhibition of autophagy may result in higher levels of autophagosomes, an analysis of the autophagic substrate p62 in parallel to LC3B-II levels is used to help resolve which process takes place. In general, an accumulation of LC3B-II and degradation of p62 reflects active autophagic flux due to an increased production of autophagosomes and greater rates of degradation of p62 in the autolysosome. Therefore, an accumulation of both LC3B-II and p62 reflects an inhibition of autophagic flux due to a failure in autophagosomal clearance and compromised lysosomal activity [15]. Figure 4G,H show that after a 72 h exposure to 40 µM HCQ, there was a much greater increase in LC3B-II and p62 levels in cells exposed to free HCQ compared to the moderate increase in cells treated with the liposomal formulations of HCQ. These changes were not observed when using the liposomal formulation with no encapsulated HCQ. In aggregate, the data shown in Figure 4 demonstrate that HCQ is effectively inhibiting autophagic flux, and the liposomal HCQ formulations appear to maintain HCQ in the liposomal form under the conditions used in these in vitro studies.

### 3.4. PK of Liposomal HCQ

The development of a liposomal formulation of HCQ was based on the idea that the liposomes would increase the circulation lifetime of the encapsulated HCQ. It was expected that over time and following the accumulation of the liposomes in sites of therapeutic interest (such as tumors), the encapsulated HCQ would be slowly released. The released HCQ would be at a concentration higher than what could be achieved with free HCQ, and this, in turn, would modulate autophagy in vivo more effectively than free HCQ. The pharmacokinetic (PK) behavior of liposomal HCQ was assessed in CD1 mice dosed IV one time with free HCQ and HCQ-loaded liposomes using the (NH_4_)_2_SO_4_ or CuSO_4_ based methods.

**Figure 5:**
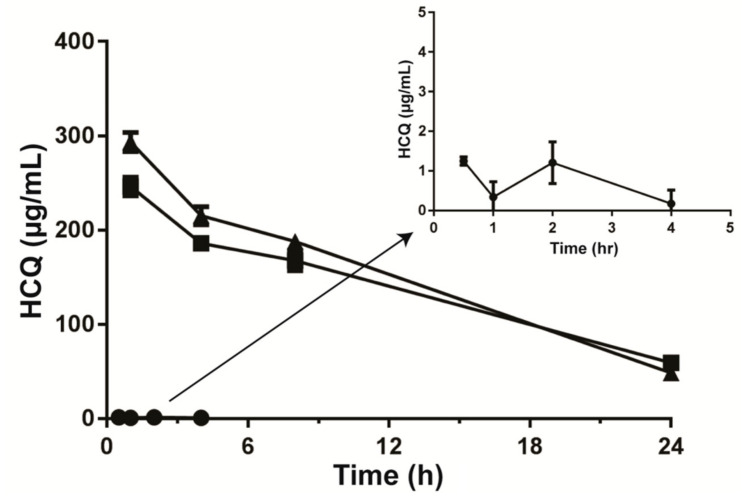
Pharmacokinetics of free HCQ and liposomal HCQ loaded in preformed (NH_4_)_2_SO_4_ or CuSO_4_ liposomes. Plasma concentration versus time profiles of free HCQ (●), HCQ in (NH_4_)_2_SO_4_-loaded liposomes (■) or HCQ in CuSO_4_-loaded liposomes (▲) administered IV at 25 mg/kg in CD-1 mice. Inset represents free HCQ using a smaller concentration scale for comparison. Plasma HCQ was measured by HPLC.

The PK data presented in Figure 5 show that free HCQ dosed IV was eliminated rapidly from the plasma compartment, with >98% of the injected dose removed within 30 min post-injection (assuming that a 22 g mouse has a plasma volume of 1 mL). The amount of HCQ in plasma after 4 h was <0.25 µg/mL. Following IV administration of the liposomal HCQ formulations, HCQ remained in circulation for extended time periods, likely as encapsulated HCQ in liposomes. It should be noted that based on the injected dose and average total plasma volume [63], about 50% of the injected HCQ was eliminated by the first time point, yet only about 12% of the injected liposomal lipid dose was eliminated. This would suggest a burst HCQ release from the liposomes within 1 h after IV injection. After 24 h, the plasma HCQ concentrations were >50 µg/mL, suggesting that about 90% of the injected HCQ was eliminated. It was estimated the liposomes remaining in the plasma compartment at this time had an HCQ-to-liposomal lipid ratio of about 0.05 (mol:mol), suggesting that 75% of the encapsulated HCQ was dissociated from the liposome 24 h after administration. Under the conditions used, the estimated AUC_0-∞_ of the free HCQ was 5.1 µg·h/mL while the (NH_4_)_2_SO_4_ and CuSO_4_ HCQ formulations exhibited estimated plasma AUC_0-∞_ values of 4360 and 4337 µg·h/mL, respectively. This reflects at least an 850-fold increase in drug exposure when using the liposomal formulations relative to free HCQ. The liposomal lipid elimination rates were essentially identical for the two formulations. It can be suggested that the release of HCQ from the different liposome formulations in vivo were comparable.

### 3.5. Efficacy of Liposome HCQ Formulations

Previously, we reported that gefitinib, a clinically relevant EGFR inhibitor, induces autophagy in various models of breast cancer in vitro [7]. These studies also showed that gefitinib in combination with HCQ engendered tumor growth inhibition in mice bearing subcutaneous HER2-overexpressing gefitinib-resistant JIMT-1 breast cancer tumor xenografts to a greater extent than either drug alone [7]. Based on the PK data presented in Figure 5 suggesting that the liposomal HCQ formulation can extend the circulation lifetime of HCQ, an efficacy study was initiated to assess if liposomal HCQ may further augment the therapeutic activity of gefitinib in vivo. The maximum feasible dose of liposomal HCQ in vivo was defined as 60 mg/kg, and at this dose, the HCQ formulation selected was well tolerated, with no sign of toxicity when given three times IV (Monday, Wednesday and Friday). The HCQ formulation selected for these in vivo studies was the one where HCQ was encapsulated in copper-containing liposomes. While the use of copper to complex HCQ made no difference in the HCQ encapsulation or HCQ release, it was postulated that the presence of copper might induce a number of different cell death mechanisms, including autophagy [52,55,57,58].

**Figure 6:**
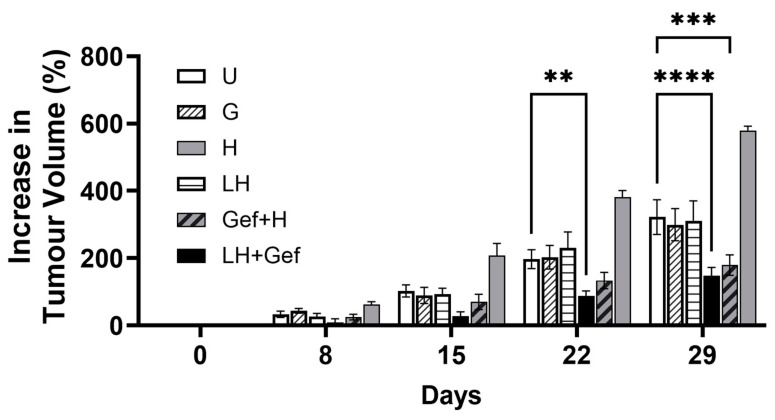
In vivo efficacy of free and liposomal HCQ in combination with gefitinib in Rag2M immunocompromised mice bearing gefitinib-resistant JIMT-1 tumors. Change in tumor volume (%) during the 4 weeks of treatment is plotted relative to the volume on the first day of treatment expressed as 0%. Data represent the mean ± SEM (n = 5–7 animals per group). U: untreated; G: gefitinib; LH: liposomal HCQ; H: free HCQ; ** *p* < 0.01, *** *p* < 0.001, **** *p* < 0.0001.

The efficacy study was completed in immunocompromised Rag2M mice bearing established JIMT-1 tumors. The animals were treated for 4 weeks with gefitinib, a liposomal CuSO_4_-based HCQ formulation and the combination of gefitinib and free or liposomal HCQ. Free HCQ was dosed IP five times a week at 60 mg/kg for a total cumulative dose of 300 mg/kg, and liposomal HCQ was dosed IV three times a week at 60 mg/kg for a total cumulative dose of 180 mg/kg. All treatments were well tolerated, and treatment-related body weight loss was less than 10% in all treatment groups. At the end of the study, neither gefitinib alone nor the liposomal HCQ formulation alone resulted in a change in tumor growth rate (Figure 6). Gefitinib in combination with free or liposomal HCQ engendered a significant decrease in tumor volume compared to untreated controls (*p* < 0.05 and *p* < 0.01, respectively; Figure 6). The efficacy of the liposomal HCQ formulation versus free HCQ when used in combination with gefitinib was not statistically different (*p* > 0.05), although the total dose of HCQ given when using the liposomal formulation was significantly lower, since it was neither feasible nor ethical to perform daily IV administration of the liposomal formulation. The results of this study were confused by the results showing that HCQ alone caused a significant increase in tumor growth when compared to the untreated mice. This enhanced tumor growth rate caused by HCQ alone has been observed in other tumor models [64]. Regardless, the results obtained for those mice treated with gefitinib and free HCQ are consistent with our previously published data and suggested significant therapeutic benefits as noted on Day 29. The combination of the liposomal HCQ formulation with gefitinib also showed therapeutic benefits, albeit the results obtained with the liposomal combination were not statistically different from the data associated with free HCQ. It can be suggested that there is a trend towards improved benefits when using the liposomal formulation, particularly when one considers that the cumulative liposomal HCQ dose was 40% lower than the cumulative free HCQ dose.

**Figure 7:**
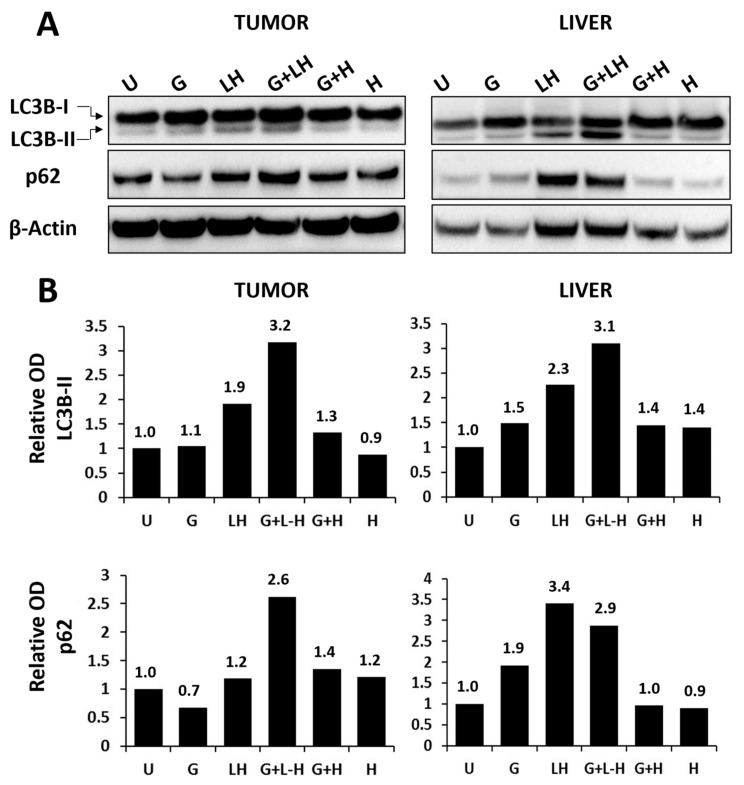
Western blot analysis of autophagy markers in JIMT-1 tumor and liver tissue derived from Rag2M mice after 4 weeks of treatment. (**A**) Western blot analysis of autophagy markers in lysates derived from tumors and livers harvested from animals four hours past the last dosing on the final day of treatment. Lysates from organs derived from 5–6 animals per group were pooled. (**B**) The expression levels of LC3B-II and p62 proteins, as shown in (**A**), were measured by densitometry and normalized to the vehicle control expressed as 1. β-actin was used to control for loading. U: untreated; G: gefitinib; LH: liposomal HCQ; H: free HCQ.

A Western blot analysis of LC3B and p62 levels in tumor tissue harvested four hours following the final dosing (Figure 7A,B, left panels) of the free HCQ (H) and gefitinib (G) alone treatment groups showed negligible changes in LC3B-II and p62 levels compared to untreated tumors. In tumors treated with the combination of gefitinib and free HCQ (G + H), there was a 1.3-fold increase in LC3B-II and a 1.4-fold increase in p62 over the controls. In tumors obtained from animals treated with the liposomal HCQ formulation alone, there was a 1.9-fold and 1.2-fold increase in LC3B-II and p62, respectively. When the tumors were obtained from animals treated with the combination of gefitinib and liposomal HCQ (G + LH) there was a 3.2- and a 2.6-fold increase in LC3B-II and p62 levels, respectively. These data suggest that liposomal HCQ may be an effective inhibitor of gefitinib-induced autophagy in vivo, unlike free HCQ, which exhibited gefitinib-induced autophagy protein changes at much lower levels (a 1.3- and 1.2-fold increase in LC3B-II and p62, respectively). Liposomal HCQ administration alone also increased LC3B-II (2.3-fold increase) and p62 (3.4-fold increase) levels in the liver compared to the controls (Figure 7A,B, right panels). Gefitinib in combination with free HCQ (G + H) caused only a 1.4-fold increase in LC3B-II and no increase in p62 levels in the liver compared to the untreated controls. In contrast, the use of liposomal HCQ and gefitinib in combination (G + LH) resulted in a 3.1-fold and a 2.9-fold increase in LC3B-II and p62 levels in liver tissue, respectively, relative to the controls. Therefore, while the Western blot assessment of LC3B-II and p62 levels in the tumor and liver suggest that the liposomal HCQ formulation in combination with gefitinib was more effective at inhibiting autophagy in vivo, the efficacy data could suggest that autophagy inhibition did not engender significantly improved therapeutic effects.

## 4. Discussion

A growing body of evidence suggests that autophagy supports cancer cell survival during metabolic and therapeutic stress, and blocking this process using genetic or pharmacological inhibitors sensitizes cancer cells to various anti-cancer agents in vitro [6,7,19,22,65]. Lysosomotropic compounds such as chloroquine (CQ) and its derivative HCQ are clinically approved anti-malarial drugs [45,48,66] that are being repurposed as autophagy inhibitors for cancer [62], with the less toxic HCQ being a preferred drug for use in humans [67,68]. Numerous clinical phase I/II trials investigating the use of HCQ in combination with various anti-cancer treatments have to date only achieved limited success. There has been no consistent treatment outcome within defined groups of patients and no noteworthy clinical gain [25,31,69]. The reasons behind these results may be due to the fact that the regulation of autophagy in different stages of cancer is not well understood, and there is no clinically validated biomarker to select patients that might be responsive to autophagy inhibition [31,69,70]. Another important factor limiting the activity of HCQ, when used as an inhibitor of autophagy, is the effective concentration that can be achieved in the blood and/or tumor site. Previously, we showed that the concentration of HCQ required to achieve a significant inhibition of autophagy in various breast cancer cells in vitro is ~20 µM, and this concentration must be maintained for several days [7]. Thus, HCQ is not very potent as an agent to inhibit autophagy, and the concentration needed in vivo is not achievable at the oral doses used as a prophylactic for malaria [71]. Pharmacokinetic studies in humans often show peak drug levels (C_MAX_) of less than 2 μM. Higher levels of HCQ can be achieved by increasing the drug dose, but these higher doses produce significant adverse events and dose-limiting toxicities such as retinopathy, myopathy, cardiomyopathy and neuromyopathy [72]. When HCQ is used as an anti-malarial agent, the reported IC_50_ values are 0.021 ± 0.002 and 2.2 ± 0.9 µM in chloroquine-sensitive (T966) and -resistant (K1) *Plasmodium falciparum*, respectively [73]. Thus, while there is a significant interest in repurposing HCQ in the clinic as part of combination regimens for the treatment of various cancer indications, its translation to clinical practice is challenging owing to differences in potency when different therapeutic targets are considered.

Since liposomes have been extensively used to enhance drug exposure, decrease plasma elimination and increase delivery to disease sites, such as tumors, all with the potential to reduce toxicity and to enhance efficacy [41,45,48,74], we investigated whether a liposomal HCQ formulation would enhance autophagy inhibition in vivo. Such a formulation was used in combination with an autophagy-promoting anti-cancer treatment, specifically gefitinib, to improve therapeutic efficacy. Others have shown that liposomes with a transmembrane pH gradient (inside acid) can be used to encapsulate HCQ through a process that is referred to as remote loading. This process has been described before for many drugs that have protonizable amine functions [50,60,75]. HCQ contains a protonizable amine (Figure 1), and the pH gradient loading method was efficient at trapping HCQ inside liposomes. HCQ has also been reported to interact with copper(II) [61]; therefore, a copper-based loading method that has been described for other drugs was also considered [23,76]. Using both (NH_4_)_2_SO_4_- and CuSO_4_-containing liposomes, the HCQ loading was efficient (>95%) and rapid, with an optimal loading temperature of 40 °C. The resulting HCQ-loaded liposomes exhibited no structural changes as assessed by cryo-EM analysis (Figure 3). In vitro studies showed that the liposomal formulations of HCQ were stable in PBS at 37 °C over 24 h, and in vitro cell-based assays completed in media supplemented with 10% FBS showed that the liposomes were not releasing sufficient levels of HCQ within 24 h to exert any significant modulation of autophagy. When the incubation time was increased to 72 h, effects were noted, albeit at lower levels than observed when using comparable concentrations of free HCQ (Figure 4). This result is consistent with the view that HCQ was stably entrapped in liposomes even in the presence of 10% serum, but these in vitro data do not predict HCQ dissociations from liposomes in vivo.

A pharmacokinetic study of the liposomal HCQ formulations demonstrated that the liposomal formulations extended the circulation lifetime of HCQ, resulting in approximately an 850-fold increase in approximate AUC_0–∞_ (Figure 5) relative to free HCQ, which was rapidly eliminated. One has to keep in mind that the plasma concentrations calculated for HCQ following the administration of liposomal HCQ are not a measure of free, but rather, total HCQ, which would include the liposomal-associated drug plus the drug that was released from the liposomes. Since free HCQ is rapidly eliminated from the plasma compartment, it can be suggested that most of the drug in the plasma compartment is liposome-associated. If there is a gradual release in HCQ from the liposomes in vivo over time, then it would be anticipated that tumor cells could be potentially exposed to higher levels of HCQ over time. The results suggest that about 75% of the liposome-associated HCQ was released over 24 h after intravenous administration.

To determine whether the liposomal HCQ formulation exhibited autophagy inhibition in vivo, studies in mice bearing tumors that arose following the subcutaneous injection of JIMT-1 breast cancer cells were completed. Previously, we showed that the in vivo treatment of HER2-overexpressing gefitinib-resistant JIMT-1 breast cancer xenografts with free HCQ and gefitinib (in combination) resulted in a 58% reduction in tumor volume compared to vehicle-treated controls [7]. If we were to evaluate the preclinical efficacy of this treatment in accordance with RECIST criteria (Response Evaluation Criteria in Solid Tumors) [77], then the treatment outcome from this previous study would fall under the category of progressive disease. Thus, it can be suggested that the treatment outcome would not be clinically relevant. The studies reported here were designed to determine if a liposomal formulation that extended the circulation lifetime of HCQ could augment the efficacy of gefitinib chemotherapy in this model. The results of this study showed that liposomal HCQ inhibited tumor growth to a similar degree to free HCQ when used in combination with gefitinib (*p* > 0.05) (Figure 6), although there was a trend to improved activity, and the liposomal drug cumulative dose was 40% lower than the free HCQ cumulative dose. It was acknowledged in the Methods that it is hard to compare the results since free HCQ was administered IP (allowing daily administration) and the liposomal formulation was given IV on a Monday, Wednesday, Friday schedule. Having indicated this, the results in Figure 6 are consistent with previous results suggesting the combination only delayed tumor progression. Another observation from the efficacy study is the unexpected increase in tumor volume with free HCQ treatment alone. While treatment with liposomal HCQ had little to no impact on tumor growth relative to the untreated controls, daily IP treatment with free HCQ was associated with worse outcomes than the untreated group. In this study, it is challenging to understand the rationale behind this observation, as free HCQ and liposomal HCQ were dosed differently. In our previous studies, free HCQ was given by oral gavage and did not accelerate tumor growth [7]. In a study completed by Wabitsch et al., HCQ treatment via oral gavage was associated with accelerated tumor growth in the MC38 colon adenocarcinoma model [64]. In that study, the authors characterized the anti-inflammatory properties of HCQ, which outweighed its speculated anti-cancer benefits in an immune-competent tumor model. Considering its interplay with the immune system, the anti-cancer mechanisms and benefits should be evaluated along with its anti-inflammatory properties if free HCQ is to be considered as part of a treatment regimen in future studies.

In the context of autophagy inhibition, the liposomal HCQ enhancement of autophagy inhibition in vivo was confirmed by an increased expression of both of the autophagic markers (LC3-IIB and p62) in tumor tissue (Figure 7A,B, left panels). In addition, there was an exceptional inhibition of autophagy in liver tissue harvested from animals at the end of treatment, a sign of efficient systemic delivery of HCQ when in a liposomal form (Figure 7A,B, right panels). Liposomes are eliminated hepatically [71,78,79], so one can expect the concentration of the liposomal drug in the liver to be much greater than that in the tumor. The modulation of autophagy in vivo is not a well-understood process, but the autophagic flux data and the PK data would suggest that exposure to HCQ released from liposomes over time is a more potent way to inhibit autophagy than exposure to a much higher HCQ concentration over a short time, as in the case of oral HCQ treatment.

One potential reason for the modest in vivo activity of the liposomal HCQ formulation used here may be due to the mildly acidic microenvironment of a tumor [80]. A lower pH would decrease the rate of HCQ release from the liposomes. To overcome this problem, Wang et al. used a targeted approach by encapsulating HCQ in liposomes coated with a pH-sensitive cationic cell-penetrating TH peptide and an RGD peptide (HCQ/Lip-TR). They showed that this formulation, in combination with liposomal doxorubicin, significantly reduced tumor growth in a B16F10 syngeneic tumor model [33]. These studies were completed in immunocompetent animals where doxorubicin is known to induce immunogenic cell death (ICD), activating an anti-tumor immune response [81,82]; thus, it is uncertain to what extent the inhibition of autophagy contributed to the overall therapeutic gain.

Based on molecular data, liposomal HCQ in combination with gefitinib appeared to inhibit autophagy in vivo more effectively than the free HCQ and gefitinib combination (Figure 6); however, these differences did not translate to improved efficacy when using liposomal HCQ in combination with gefitinib. As already indicated, the experiments were designed in a manner where the free drug was given at a total cumulative dose higher than what was used with the liposomal formulation. It would be worthwhile to enhance the dose intensity and explore different lipid compositions of the HCQ formulation, to assess how HCQ release rate from the liposomes following administration may affect the inhibition of autophagy in vivo and therapeutic activity when used in combination with autophagy-inducing agents. The results in Figure 6 clearly indicate that at the liposomal HCQ dose used, there was no impact on tumor progression when used alone. If this approach were pursued clinically, it does raise an interesting development question. If the new agent (liposomal HCQ) has no single-agent activity for a cancer indication, then it would be ethical to use it as a clinical candidate in a phase I dose escalation study in cancer patients.

This research suggests that a liposomal formulation of HCQ could be used to inhibit autophagy in vivo. The formulation, when used in combination with gefitinib, did reduce the rate of tumor growth, and the effects were better, albeit not significantly, to what was observed when using free HCQ. The molecular data did suggest that autophagic flux was inhibited more when using the liposomal HCQ formulation. Together, the results highlight the complexity of the molecular mechanisms responsible for modulating autophagy in vivo and whether an effective modulation of autophagy will improve the anti-cancer activity of autophagy-promoting agents. HCQ is not a specific inhibitor of autophagy, and many questions remain unanswered regarding the mechanistic interactions between autophagy-inducing drugs and HCQ, especially in the context of tumor-specific molecular pathways and the off-target effects of HCQ that are contributing to the overall pharmacological activity of this drug [25,72,83,84,85,86]. The results presented here encourage further research focusing on optimizing liposomal formulations of HCQ for in vivo use. Liposomal formulations of new potent autophagy inhibitors that target autophagosome formation such as verteporfin, autophagy-related 4 cysteine peptidase (ATG4) inhibitors or ULK1 inhibitors should be considered [31,62,87,88,89]. As autophagy has been implicated in supporting ICD-inducing anti-cancer immune responses [25,90,91], careful consideration should also be given to completing efficacy studies in immune-competent mice.

## Figures and Tables

**Table 1:** Characteristics of HCQ-encapsulated DSPC/Chol liposomes.

Formulation	Particle Size (nm)	Polydispersity
(NH_4_)_2_SO_4_ Liposomes	124 ± 0.8	0.122
HCQ-(NH_4_)_2_SO_4_	114 ± 1.5	0.09
CuSO_4_ Liposomes	101 ± 1.5	0.038
HCQ-CuSO_4_	106 ± 0.7	0.126

## Data Availability

The original contributions presented in the study are included in the article, further inquiries can be directed to the corresponding author.
